# Standardized Protocol Items Recommendations for Observational Studies (SPIROS) for Observational Study Protocol Reporting Guidelines: Protocol for a Delphi Study

**DOI:** 10.2196/17864

**Published:** 2020-10-21

**Authors:** Raman Mahajan, Sakib Burza, Lex M Bouter, Klaas Sijtsma, André Knottnerus, Jos Kleijnen, Peter Van Dael, Maurice P Zeegers

**Affiliations:** 1 Care and Public Health Research Institute Maastricht University Maastricht Netherlands; 2 Médecins Sans Frontières New Delhi India; 3 Department of Epidemiology and Data Science Amsterdam University Medical Centers Amsterdam Netherlands; 4 Department of Philosophy Vrije Universiteit Amsterdam Netherlands; 5 Department of Methodology and Statistics Tilburg School of Social and Behavioral Sciences Tilburg University Tilburg Netherlands; 6 DSM Nutritional Products Basel Switzerland; 7 School for Nutrition and Translational Research in Metabolism Maastricht University Maastricht Netherlands

**Keywords:** protocol, observational studies, SPIROS, guidelines, Delphi

## Abstract

**Background:**

Approximately 90% of currently published clinical and public health research is in the form of observational studies. Having a detailed and registered study protocol prior to data collection is important in any empirical study. Without this, there is no reliable way to assess the occurrence of publication bias, outcome reporting bias, and other protocol deviations. However, there is currently no solid guidance available on the information that a protocol for an observational study should contain.

**Objective:**

The aim of this study is to formulate the Standardized Protocol Items Recommendations for Observational Studies (SPIROS) reporting guidelines, which focus on 3 main study designs of analytical epidemiology: cohort, case-control, and cross-sectional studies.

**Methods:**

A scoping review of published protocol papers of observational studies in epidemiology will identify candidate items for the SPIROS reporting guidelines. The list of items will be extended with the Strengthening the Reporting of Observational Studies in Epidemiology (STROBE) checklist items and recommendations from the SPIROS steering committee. This long list serves as the basis for a 2-round Delphi survey among experts to obtain consensus on which items to include. Each candidate item from the long list will be rated on a 5-point Likert scale to assess relevance for inclusion in the SPIROS reporting guidelines. Following the Delphi survey, an expert-driven consensus workshop will be convened to finalize the reporting guidelines.

**Results:**

A scoping review of published observational study protocols has been completed, with 59 candidate items identified for inclusion into the Delphi survey, itself launched in early 2020.

**Conclusions:**

This project aims to improve the timeliness, completeness, and clarity of study protocols of observational studies in analytical epidemiology by producing expert-based recommendations of items to be addressed. These reporting guidelines will facilitate and encourage researchers to prepare and register study protocols of sufficient quality prior to data collection in order to improve the transparency, reproducibility, and quality of observational studies.

**International Registered Report Identifier (IRRID):**

PRR1-10.2196/17864

## Introduction

The protocol of any empirical study offers the researcher practical guidance in data collection, analysis, and reporting. It also forms the basis for subsequent replication of the study and detection of publication bias, outcome reporting bias, and other protocol deviations. A well-formulated study protocol uploaded prior to data collection through a time-stamped registry will allow a robust audit trail. This improves the chances that the study is appropriately planned, executed, and well documented, thus promoting optimal conduct, accountability, replicability, and overall research integrity. In summary, ensuring a high level of transparency in the research planning phase facilitates replicability of the study and fosters good research practices [1,2].

Although the value of registered study protocols and reporting guidelines on how to write them are widely recognized in the field of randomized control trials (RCTs) [3-5], reporting guidelines for study protocols of observational studies in analytical epidemiology (cohort, case-control studies, and cross-sectional studies) are currently absent. In many situations, observational studies may be the best or at least the only feasible way to answer important medical and public health questions in scenarios where RCTs are not possible for practical and ethical reasons. Therefore, the same rigorous standards that apply to RCTs must be maintained for observational studies.

An estimated 90% of currently published clinical or public health–related research is in the form of observational studies [6]. In particular, most research focusing on understanding causes and distribution of diseases relies on cohort, case-control, or cross-sectional studies. Additionally, a substantial body of evidence for diagnostic or prognostic research takes the form of observational studies. The absence of reporting guidelines on how to write these study protocols is problematic, as by design, observational studies have a nonnegligible risk of bias [7]. The risk of bias has been well recognized in experimental research, where guidelines for the development of RCT protocols are well established [1]. Additionally, large observational data sets without any statistical analysis plan readily allow data-driven post hoc analyses, leading to an exceedingly large number of potential associations that can be tested [8,9]. In such situations of multiple comparisons, there is increased likelihood of type I error and “p-hacking” by doing multiple statistical tests on the data and only reporting those that come back with significant results, causing false-positive results [10]. Therefore, it is important to ensure that study protocols are complete, well written, and registered prior to data collection in a suitable time-stamped repository (eg, ClinicalTrials.gov and Open Science Framework). This allows external bodies to consult the original study protocol and understand the specific hypotheses and the data analysis plan to which the researcher was committed, both for primary data analyses and for secondary analyses of existing data sets.

When protocols of observational studies do not provide sufficient details concerning the conduct of their preplanned study and are not preregistered or made openly available prior to data collection initiation, the scientific community is left with an incomplete picture of what the researchers intended to do and whether undisclosed protocol deviations and reporting biases occurred. Consequently, in the absence of registered protocols for observational studies, we are insufficiently able to judge the validity and reliability of the results. As such, interpretation of the results within the context of the study limitations becomes a potential major issue [2].

## Methods

### Overview of Objectives

The objective of this study is to develop reporting guidelines to be used in the design of observational study protocols. Our initiative has been given the acronym SPIROS (Standardized Protocol Items: Recommendations for Observational Studies). The project aims to improve the completeness and utility of study protocols for observational studies by producing expert-based recommendations for a minimum set of items these study protocols need to address. An item is defined as an important section, heading, or subheading within the study protocol, with each item describing an important methodological feature of the study. Our study is designed to meet three objectives.

Objective 1 is to conduct a scoping review of items addressed in study protocols of observational studies in epidemiology. The purpose of objective 1 is to develop a long list of candidate items that could a priori be incorporated in the draft reporting guidelines. This would serve as the basis for the first round of the Delphi survey (Objective 2).

Objective 2 is to conduct a Delphi survey with participants who are selected based on a prespecified range of expertise, with the intention of obtaining consensus on which items to include in the SPIROS reporting guidelines.

Objective 3 is to conduct a face-to-face consensus workshop to finalize the SPIROS reporting guidelines and then disseminate the results with an associated manual.

### Methods to Conduct Scoping Review of Protocols (Objective 1)

A scoping review of published study protocols of observational studies will be conducted, in which every item mentioned more than once will be listed [11,12]. The study will be based on a systematic selection of up to 100 protocols of observational studies identified via published study design papers indexed by Web of Science Core Collections until information threshold saturation is achieved (defined as the point where no further items are identified after 15 consecutive paper reviews). The list of items will then be extended with the Strengthening the Reporting of Observational Studies in Epidemiology (STROBE) checklist items, which are developed as reporting guidelines for the publication of observational studies [13], the Standard Protocol Items: Recommendations for Interventional Trials (SPIRIT) checklist items for the reporting of protocols of experimental studies [3], and recommendations from an expert steering committee (the authors of this manuscript). The findings of the scoping review will be reported according to the Preferred Reporting Items for Systematic Reviews and Meta-Analyses (PRISMA) extension for scoping reviews [14].

#### Eligibility Criteria

Up to 100 protocols of observational studies (cohort studies, case-control studies, and cross-sectional studies) published in indexed journals from the Web of Science will be included in the present review. To ensure the most current state of affairs is assessed, only papers published between January 1, 2016, and May 1, 2018, will be included because, based on a preliminary assessment, the target number of studies is expected to be reached within this time range.

#### Information Sources

Data for this scoping review will be identified by searching the Web of Science Core Collection and references from relevant papers using the search terms “protocol” PLUS “observational study” AND/OR “cohort study” AND/OR “case control study” AND/OR “cross sectional study” AND/OR “prevalence study” AND/OR “survey” in the advanced search mode. A senior clinical librarian will be engaged to ensure that the search strategy is valid.

All the protocol items present in the headings, subheadings, or text of protocol papers will then be presented in the form of a table, following the structure of the STROBE checklist. Initially, all items will be categorized into the following themes: general information, introduction, methods, ethical considerations, reporting, dissemination, and others. However, if different themes emerge, this structure will be accordingly iteratively adapted.

### Methods to Conduct Delphi Study (Objective 2)

#### Participant Recruitment

The long list of candidate items for the protocols of observational studies identified in the scoping review will be evaluated by experts. For this purpose, we will conduct a 2-round Delphi study [15-19]. We will aim for participation of 50 to 100 experts who represent the key stakeholders in observational research, including principal investigators, methodologists, journal editors, users of observational data, and representatives from research ethics review boards. Experts will need to have relevant knowledge and experience and be spread across geographical regions. Experts will be identified by a mixed approach, including nomination by other experts, PubMed and web search, ResearchGate, and participation in the development of previous reporting guidelines.

#### Round 1 of Delphi Survey

Each panelist will be asked to rate every candidate item from the long list on a 5-point Likert scale for relevance for inclusion in the reporting guidelines for protocols of observational studies (Table 1).

A field for adding free text will be provided for comments on comprehensiveness and comprehensibility for each item and to suggest alterations or additional items. Round 1 will also collect core demographic information (panelists’ working field and place of employment) and panelists’ self-rated level of expertise on the particular topic.

A threshold of 67% agreement among participants on a score of 4 or 5 for each candidate item will be considered to indicate consensus among the expert panel for consideration of inclusion in the SPIROS checklist. If agreement among participants is less than 67% scoring 4 or 5, the item will be considered discordant. The survey will be structured to last no more than 30 minutes and will be pretested and approved by the expert steering committee. The study protocol with any amendments will be uploaded in the Open Science Framework (OSF), as will the Delphi survey format, which will be updated, if needed, before the second round.

**Table 1:** Implication of Likert score on candidate items.

Likert score	Implication on candidate item
1	Unimportant—should be dropped as an item to consider
2	Of little importance
3	Moderately important
4	Important
5	Very important—should definitely be included

#### Round 2 of Delphi Survey

Round 2 will contain all original candidate items plus additional items nominated or identified during the first round. For each item, panelists will be provided with percentage agreement from the first round, while the themes of each item´s free text comments will be merged into an anonymized executive summary.

The participants will be asked to rerate the items and consider the comment themes. Once collated, all items reaching a 67% consensus agreement ranking of 4 or 5 on the Likert score will be included in the short list for the reporting guidelines and advance to the next stage of the process.

#### Data Collection and Statistical Analysis

A preset online survey questionnaire will be developed on the Google Forms online survey platform and uploaded to OSF before being sent out to expert panelists via email. Medians, interquartile range, and percentage of agreement will be calculated for each item.

### Consensus Workshop (Objective 3)

Following completion of the Delphi survey, the steering committee will convene a consensus workshop consisting of the steering committee plus 10 others previously involved in developing guidelines. Experts will review and adapt the draft reporting guidelines into a format appropriate for wider dissemination. A further workshop around dissemination strategy planning will also be convened, comprising experts involved with previous guideline dissemination, to ensure that a robust uptake mechanism is defined for widespread adoption of SPIROS, including choice of journal for publication of the final manuscript and content of a website exclusively for the initiative.

### Dissemination, Implementation, and Ongoing Development (Objective 4)

The ultimate objective of this initiative is to facilitate widespread adoption of the SPIROS reporting guidelines. Therefore, all outputs of this initiative will be published as preprints and in open access journals or open choice access. This will include a detailed manual that explains and justifies the recommendations and includes examples of good reporting. We aim to have the SPIROS reporting guidelines adopted by journals and funders. The SPIROS protocol and final reporting guidelines will be published on a dedicated website (spiros-statement.org) and will be included in the Enhancing the Quality and Transparency of Health Research (EQUATOR) Network.

### Data Management and Registration of Study Protocol

The study will comply with the EU General Data Protection Regulation. Prior to data collection, the study protocol and all appendices will be registered and made openly available on the OSF, and links will be provided to reviewers and readers of the paper. This will include the complete data management and data analysis plan, and on completion of the study, all anonymized data will additionally be made available on OSF with no restrictions.

### Ethics Approval and Consent to Participate

Ethics approval has been obtained from the Ethics Review Committee of Maastricht University. The study will not collect any sensitive information, and the identity of each panel member will be fully protected and blinded to other panel members and the research group (except for the corresponding researcher). The burden for the panelists includes a maximum of 30 minutes for each of the Delphi survey rounds.

Online informed consent will be obtained from all panel members, which will comprise an introductory page and completion of the survey as consent. Participants of the Delphi consensus survey will be provided with a summary of the overall aim of the SPIROS project and a link to the complete protocol. The consent for the Delphi survey will explain the specific aim of the survey and an outline of the procedures involved, as well as the benefits, risks, and burdens involved in participating. If consent is given, panels member will be acknowledged in the publication of the SPIROS reporting guidelines. However, the option to not be mentioned in the acknowledgments will also be given.

## Results

The scoping review of published protocols of observational studies has been completed, with 59 candidate items identified. This long list of candidate items has formed the basis of the 2-round Delphi survey launched in early 2020.

## Discussion

Over the last decade, there has been an increasing call for prospective registration of observational study protocols [6,20-22]. Without a registered protocol, it is impossible to detect p-hacking and any intentional amendments in protocols to match with preferred results. To conduct research without a detailed protocol registered prior to data collection is increasingly considered questionable research practice, limiting reviewers’ and readers’ ability to assess the occurrence of intentional or nonintentional bias. A detailed study protocol is essential for replicability as well. That said, even with strict prospective registration of observational study protocols, including digital object identifier records of any amendments, there remains the chance of post hoc protocol amendments at a later stage to match with preferred results. Ultimately, there is no foolproof way to prevent scientific malpractice in this domain.

There are several recent projects, such as the Reproducible Evidence: Practices to Enhance and Achieve Transparency (REPEAT) initiative, that aim to improve transparency, reproducibility, and validity of database research [23]. At the same time, the need for one-size-fits-all guidance, or any guidance for protocol development of observational studies, remains a subject of debate [20,21,24,25]. For example, an industry-based epidemiological group have proposed a framework in which observational studies should be registered and can be exempted based on study design and study intent [25].

However, the absence of guidelines to develop protocols has historically resulted in a large proportion of observational research being conducted without quality-assured study protocols. Existing protocols are often not fit for purpose, and the proportion of researchers ensuring registration of protocols for observational studies prior to data collection remains negligible. Therefore, our study aims to develop reporting guidelines for protocols of observational studies by launching SPIROS. We expect this initiative to increase transparency of methods used in observational studies, and through standardizing protocols, we hope that study designs will be improved and more often be registered and published in a journal or in a repository.

The main limitation of our study is that SPIROS reporting guidelines will be developed primarily for cohort, case-control, and cross-sectional study designs but not for other observational research designs. Because of the wide scope of observational study designs, it is difficult to prepare standard reporting guidelines that cover all observational study designs.

Nevertheless, as the first attempt to develop a guiding document for observational study protocols, our guidelines will fill an important gap. Once widely available, an impact assessment should be conducted after a 4- to 5-year period to determine whether more and higher-quality protocols have become available, similar to the approach adopted by other reporting guidelines [26].

## Abbreviations

EQUATOR: Enhancing the Quality and Transparency of Health Research
OSF: Open Science Framework
PRISMA: Preferred Reporting Items for Systematic Reviews and Meta-Analyses
RCT: randomized control trial
REPEAT: Reproducible Evidence: Practices to Enhance and Achieve Transparency
SPIRIT: Standard Protocol Items: Recommendations for Interventional Trials
SPIROS: Standardized Protocol Items: Recommendations for Observational Studies
STROBE: Strengthening the Reporting of Observational Studies in Epidemiology
